# Insight into a single-chamber air-cathode microbial fuel cell for nitrate removal and ecological roles

**DOI:** 10.3389/fbioe.2024.1397294

**Published:** 2024-07-08

**Authors:** Xiaojun Jin, Nuan Yang, Dake Xu, Cheng Song, Hong Liu

**Affiliations:** ^1^ Shenyang National Laboratory for Materials Science, Northeastern University, Shenyang, China; ^2^ CAS Key Laboratory of Reservoir Aquatic Environment, Chongqing Institute of Green and Intelligent Technology, Chinese Academy of Sciences, Chongqing, China; ^3^ Key Laboratory of Development and Application of Rural Renewable Energy, Ministry of Agriculture and Rural Affairs, Biogas Institute of Ministry of Agriculture and Rural Affairs (BIOMA), Chengdu, China

**Keywords:** microbial fuel cells, nitrate removal, nutrient distribution, denitrifying bacteria, electroactive denitrifying bacteria

## Abstract

Bioelectrochemical systems are sustainable and potential technology systems in wastewater treatment for nitrogen removal. The present study fabricated an air-cathode denitrifying microbial fuel cell (DNMFC) with a revisable modular design and investigated metabolic processes using nutrients together with the spatiotemporal distribution characteristics of dominated microorganisms. Based on the detection of organics and solvable nitrogen concentrations as well as electron generations in DNMFCs under different conditions, the distribution pattern of nutrients could be quantified. By calculation, it was found that heterotrophic denitrification performed in DNMFCs using 56.6% COD decreased the Coulombic efficiency from 38.0% to 16.5% at a COD/NO_3_^−^-N ratio of 7. Furthermore, biological denitrification removed 92.3% of the nitrate, while the residual was reduced via electrochemical denitrification in the cathode. Correspondingly, nitrate as the electron acceptor consumed 16.7% of all the generated electrons, and the residual electrons were accepted by oxygen. Microbial community analysis revealed that bifunctional bacteria of electroactive denitrifying bacteria distributed all over the reactor determined the DNMFC performance; meanwhile, electroactive bacteria were mainly distributed in the anode biofilm, anaerobic denitrifying bacteria adhered to the wall, and facultative anaerobic denitrifying bacteria were distributed in the wall and cathode. Characterizing the contribution of specific microorganisms in DNMFCs comprehensively revealed the significant role of electroactive denitrifying bacteria and their cooperative relationship with other functional bacteria.

## 1 Introduction

Removing nitrate from wastewater is essential for preventing the pollution of receiving water bodies because nitrate can cause water eutrophication and pose a health threat to animals and humans. Biological treatment is generally less expensive in terms of maintenance and operation costs than chemical and physical processes. Microbial fuel cells (MFCs), especially single-chamber MFCs (SCMFCs), not only benefit from direct electricity generation and reduced sludge production but also save aeration energy and enhance nutrient removal (Yan et al., 2012; Yang et al., 2020). Therefore, SCMFCs have become a potential technology for nitrogen removal in wastewater treatment (Liu et al., 2019; Yang and Cheng, 2019).

Recently, a nitrogen removal mechanism in the single-chamber MFCs has been proposed based on microbial community analysis. In the first phase, ammonium is oxidized to nitrite/nitrate by nitrifying bacteria, which are distributed on the surface of the cathode in a micro-aerobic environment (Yang et al., 2021; Gurung et al., 2023). Next, nitrification products are either reduced through heterotrophic denitrification by denitrifying bacteria (DNB) (Huang et al., 2018) or via bioelectrochemical denitrification by electroactive bacteria (EAB) (Qiao et al., 2018). The main pathway of heterotrophic denitrification in MFCs is consistent with conventional nitrification and denitrification, whereas bioelectrochemical denitrification is a novel autotrophic denitrification, wherein nitrate as the electron acceptor is reduced to N_2_ or NH_3_ in the biocathode of denitrifying MFCs (DNMFCs) (Zhang and He, 2012; Zhang et al., 2020).

Dominated microorganisms play an important role in DNMFC performance, which involves the degradation of organic matter and nitrogen. Considering the microbial community structure, with the increase in the nitrogen concentration, DNB gradually dominated the DNMFCs instead of the original EAB (Huang et al., 2018), and the performance of electricity generation was hardly affected by the abundance of organics. For example, *Geobacter*, the most prevalent EAB in acetate-fed systems, barely appeared in the DNMFC biofilms. However, bifunctional bacteria with abilities of electroactivity and biological denitrification, such as *Thauera* (Yang et al., 2019) and *Pseudomonas* (Huang et al., 2018), played a key role in nitrogen removal and power recovery. Compared to the DNB without electroactivity, these electroactive DNB (EDNB) with high abundance simultaneously perform complete denitrification and electron transfer and are generally unaffected by the C/N ratios in MFCs (Jin et al., 2022). Hence, the nitrogen removal mechanism in DNMFCs appears to be considerably more complex than that of traditional biological denitrification. To date, the classification and distribution of specific microorganisms in DNMFCs are still controversial, necessitating further investigation to provide insight into the nutrient-removal mechanism of DNMFCs.

Therefore, this study focused on the comprehensive analysis of nutrient removal and the dominant specific microorganisms in a denitrifying air-cathode single-chamber MFC. We fabricated a DNMFC to calculate the distribution of acetate and nitrogen; meanwhile, the specific functional microorganisms were also analyzed based on the microbial community analysis. Quantifying the nutrient distribution and the contribution of functional microorganisms in DNMFCs can effectively guide the efficient removal of nitrogen-containing wastewater.

## 2 Materials and methods

### 2.1 MFC configuration

This study used six air-cathode single-chamber MFCs consisting of two identical chambers (a Plexiglas cylinder, 2 cm in length and 3 cm in diameter) with a total working volume of 30 mL. The anode consisted of a circular carbon cloth (W0s1009, Phychemi Co. Ltd., China) with a diameter of 3.0 cm. A commercial carbon cloth (W1s1009, Phychemi Co. Ltd., China) laminating a blocking layer of active carbon on one side was used to prepare the air-cathode (Gao et al., 2021). Four poly(dimethylsiloxane) (PDMS, Sylgard 184, Dow Corning, Auburn, MI) layers brushed on the filled side were considered diffusion layers exposed to the air, while Pt/C of 0.5 mg/cm^2^ coated on the other side was regarded as a catalyst layer exposed to the solution (Zhang et al., 2010). The anode and cathode were placed on opposite sides of each chamber and connected to an external resistor of 1kΩ using a titanium wire.

### 2.2 MFC startup and operation

The startup of DNMFCs was shortened by hanging the anodes on a mature anode in the long-term, stable, large-scale MFC beforehand for more than 1 month. As the inoculum, the composition of the anodic microbial community in the anode chamber of the dual-chambered MFC is shown in Supplementary Figure S1. The pre-enrichment stage lasted 35 days (∼5 batches). The synthetic wastewater containing 1.0 g/L CH_3_COONa (∼700 mg/L COD), 10.13 g/L Na_2_HPO_4_, 6.08 g/L NaH_2_PO_4_, 0.13 g/L KCl, 0.71 g/L NaNO_3_ (96 mg/L NO_3_^−^-N), and 12.5 mL/L Wolfe’s mineral solution was fed. The COD/NO_3_^−^-N ratio of wastewater was approximately 7, which was purged with N_2_ for 30 min s before injecting into the reactor. The reactors were operated in a fed-batch mode at an ambient temperature of 30°C ± 1°C.

**FIGURE 1:**
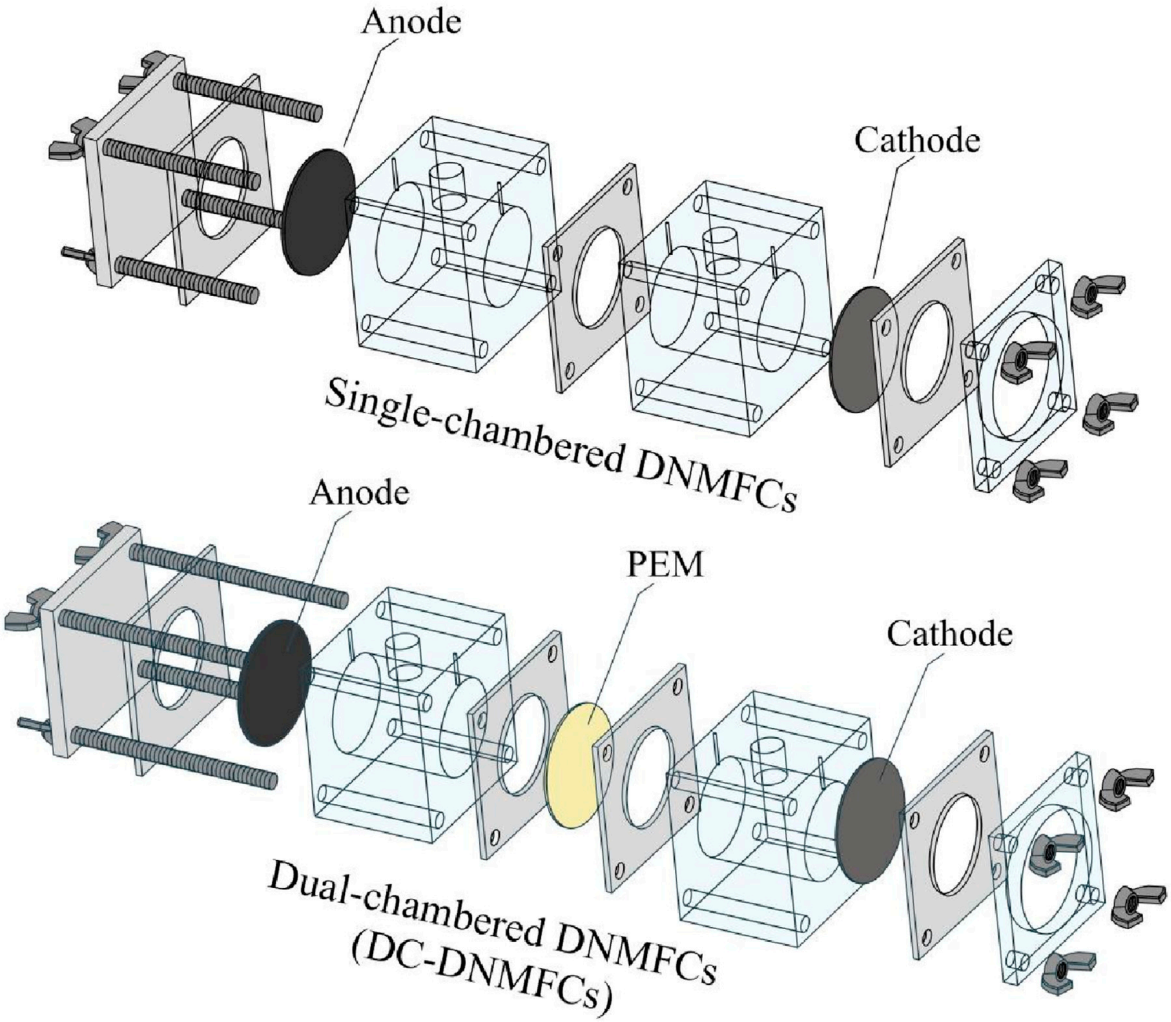
Schematic diagram of DNMFCs and modification from a single chamber to dual chambers.

After stable operation, a series of tests were conducted (such as organics removal, electron recovery, and nitrogen detection) to evaluate electricity generation and nitrogen conversion. For further analysis of special microorganisms, the distribution of DNB, and their contribution to DNMFCs, the original single chamber was divided into two identical chambers after 50 days of stable operation, which were separated by a proton exchange membrane (Nafion 117, Dupont) (Figure 1). The DNMFCs with single-chamber and dual-chamber were termed DNMFCs and DC-DNMFCs, respectively. Other parameters remained unchanged without special instructions, and various parameters affecting nitrate reduction were investigated. All tests were conducted in triplicate, and parallelization of more than three cycles was tested under identical conditions in each test to ensure the accuracy of the results.

### 2.3 Analysis and calculations

During the MFC batch operation stage, effluent samples were withdrawn from each batch using a syringe and passed through a syringe filter (0.45 μm pore diameter) before analysis. The concentrations of chemical oxygen demand (COD), NH_4_^+^-N, NO_2_^−^-N, and NO_3_^−^-N were measured according to the APHA standard methods (APHA, 2001). The output voltages were recorded at 5-min intervals using a computer with a data acquisition system, as described previously (Jin et al., 2022). Polarization and power density curves were calculated by varying the external resistor from 10 to 0.01㏀. The generated electric charge (Ee, Q), theoretical consumed electric charge of nitrate conversion (E_N_, Q), theoretical generated electric charge of acetate (E_A_, Q), Coulombic efficiency (CE, %), and electron transfer efficiency (ETE, %) were calculated using Eqs 1–5:

$$\text{Ee}=\int_{0}^{t}Idt,$$ (1)

$$E_{N}=\frac{F\times V\times ∆C_{N}\times 5}{14},$$ (2)

$$E_{A}=\frac{F\times V\times ∆COD\times 4}{32},$$ (3)

$$CE=\frac{Ee}{E_{A}}\times 100,$$ (4)

$$\text{ETE}=\frac{\text{Ee}+E_{N}}{E_{A}}\times 100,$$ (5)

where I is the current, t is the time, F is the Faraday’s constant (96,485 Q/mol), V is the volume (30 mL), ΔC_N_ is the amount of NO_3_^−^-N reduced, and ΔCOD is the amount of COD consumed.

Throughout the process, a commercial DO microelectrode (Unisense, Denmark) was separately inserted in the middle of the anode and cathode chambers to measure the DO value. Before each measurement, the microelectrode was calibrated following the manufacturer’s instructions. Independent triplicate experiments were conducted for each stability test.

### 2.4 Micromorphology observation and microbial community analysis

The micromorphology of the electrode biofilms was observed using a scanning electron microscope (SEM) (JSM-6510LV, Japan) and a confocal laser scanning microscope (CLSM) (inVia Reflex, UK). Before SEM testing, the samples were pretreated as follows: (1) the anode samples were immersed for 4 h in the fixative containing 2.5% glutaraldehyde at 4 °C; (2) the samples were rinsed with 0.1 mol/L PBS solution; (3) they were then dehydrated with 50, 70, 80, and 90% ethanol, respectively; and (4) they were further dehydrated with tert-butyl alcohol; and (5) finally, they were tested after being coated with a 10 nm layer of gold to reduce charging during SEM analysis. Before CLSM testing, the samples were pretreated as follows: (1) the anode samples were rinsed with 0.1 M PBS solution to eliminate the original medium; (2) stained using a LIVE/DEAD BacLight Viability Kit (L13152, Life Technologies, USA) for 20 min; and (3) rinsed with 0.1 M PBS solution to eliminate excess dye. A CLSM with ×20 objective was used to visualize the spatial live/dead topography of the biofilms.

Total genomic DNA was extracted from an entire piece of each electrode, and the bacterial 16S rDNA was PCR-amplified using primers 338F and 806R targeting the variable V3–V4 region (forward primer: 5′-CCT​ACG​GGA​GGC​AGC​AG-3′; reverse primer: 5′-GGACTACHVGGGTWTCTAAT-3′) (Jin et al., 2022). The PCR products were sequenced using the MiSeq Illumina platform (Majorbio Bio-pharm Technology, Shanghai, China). These data analyzed were used to express the microbial community distribution at the class and genus levels.

## 3 Results and discussion

### 3.1 Electrochemical characteristics and nitrogen removal of DNMFCs

**FIGURE 2:**
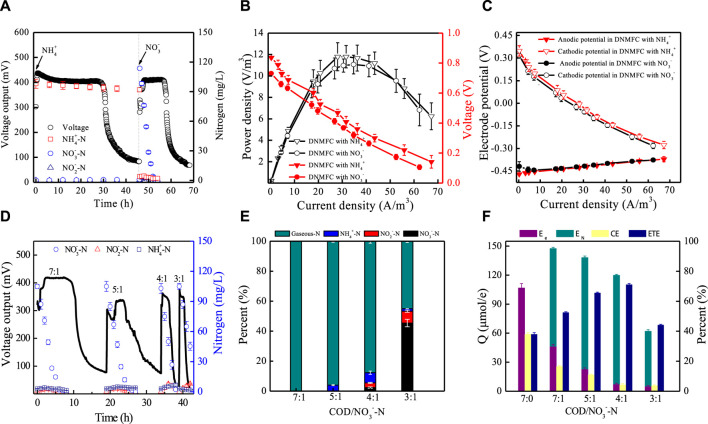
Properties of single-chambered DNMFCs in nitrogen removal and electricity performance. **(A)** Voltage outputs and nitrogen concentration changes in DNMFCs with NH_4_^+^/NO_3_^−^ containing wastewater treatment. **(B,C)** Power densities and electrode polarization curves. **(D–F)** Electron and nitrogen transfer in DNMFCs with different C/N ratios. E_e_, generated electric charge; E_N_, theoretical consumed electric charge of nitrate conversion; CE, Coulombic efficiency; ETE, electron transfer efficiency.

Ammonium was used instead of nitrate to compare the electricity performance and nitrogen-removal abilities of DNMFCs treating NH_4_^+^- and NO_3_^−^-containing wastewater (Figures 2A–C). First, NH_4_^+^ loss in DNMFCs was only 6.2%, and null concentration of NO_2_^−^ and NO_3_^−^ was detected in the bulk solution throughout the cycle operation, indicating that ammonium oxidation did not occur on the air-cathode surface (Figure 2A). It means that the nitrifying process does not contribute to organic oxidation and electron flux and that the nitrifying bacteria do not participate in the oxygen and nitrate reduction reactions. Both the pre-enrichment of anode biofilm and the high activity of air-cathode with Pt/C maintained a low DO concentration in the solution even during startup, which finally inhibited the occurrence of nitrification in this system.

When DNMFCs were treated with nitrate-containing wastewater, NO_3_^−^ was rapidly reduced with no noticeable accumulation of intermediates during the entire process, demonstrating complete biological denitrification by DNB (Yang et al., 2019). Second, the cycle time of MFCs in the presence of NH_4_^+^ was approximately two times longer than that of NO_3_^−^. Therefore, the relative electron recovery of DNMFCs sharply decreased from 106.8 ± 0.3 to 46.7 ± 0.2 μmol/e, respectively (Figures 2A, F). Furthermore, similar waves of power density and polarization curves of DNMFCs with ammonia- and nitrate-containing wastewater treatment indicated that the appearance of stable and mature biofilms on the electrodes was not affected by nitrogen composition.

According to polarization curves, the anode potentials in MFCs were highly consistent with the increase in current densities, attributed to the pre-enriched biofilm of the anodes before the reactor startup. The cathode potential also tended to be stable, suggesting that the microbial community in the electrode biofilms of DNMFCs strongly tolerated nitrogen stress. Therefore, the method for DNMFC startup in this study is successful and efficient. The cathode biofilm in the DNMFCs displayed an increased EDNB abundance, which partly compensated for the active site reduction of Pt/C. The oxygen reduction reaction (ORR) by the chemical catalyst of Pt/C can be performed in a series of reactions with the cooperation of Pt/C, DNB, and EAB. Compared to the metal-free carbon cloth used as the cathode, the metal-coated cathode in SCMFCs has a shorter startup time, higher bioelectrochemical activity, and higher nitrogen removal efficiency (Xin et al., 2020; Yan et al., 2012; Yang et al., 2019).

In DNMFCs, the electrochemical activity and performance of electricity generation are closely related to the ratios of COD/NO_3_^−^-N in wastewater. As shown in Figures 2D–F, the electron fluxes were further investigated at COD/NO_3_^−^-N ratios from 7 to 3, and high efficiencies of electrochemical activity and nitrogen removal were obtained in DNMFCs. NO_3_^−^ was majorly removed when the COD/NO_3_^−^-N ratio was above 4, which is close to the value of 3.5 for nitrate reduction by heterotrophic denitrifying bacteria (Knowles, 1982) and much lower than that of 10 in practical wastewater treatment for nitrogen removal (Sun et al., 2010). Furthermore, compared to general heterotrophic denitrification, electrochemical techniques for nitrate removal have the advantages of high efficiency, active sludge decrement, and lack of additional chemical reagent addition. However, CEs sharply declined with the decrease in the COD/NO_3_^−^-N ratio, indicating that the nitrate reduction reaction is very important and should be unignored in DNMFCs. As shown in the results, the rate of nitrate reduction is much quicker than that of other reduction reactors. Therefore, electron transfer efficiency seems much more reasonable than characterizing the performance of DNMFCs. In DNMFCs, ETEs increased from 52.5% ± 0.5% to 71.1% ± 0.8% and then decreased to 44.2% ± 0.5% at a COD/NO_3_^−^-N ratio of 3. Under the condition of insufficient organic matter, the intermediate of NH_4_^+^ considerably accumulated, suggesting the presence of electrochemical nitrate reduction to ammonia in DNMFCs.

### 3.2 Deep insight into the contribution of nitrate reduction in DNMFCs

**FIGURE 3:**
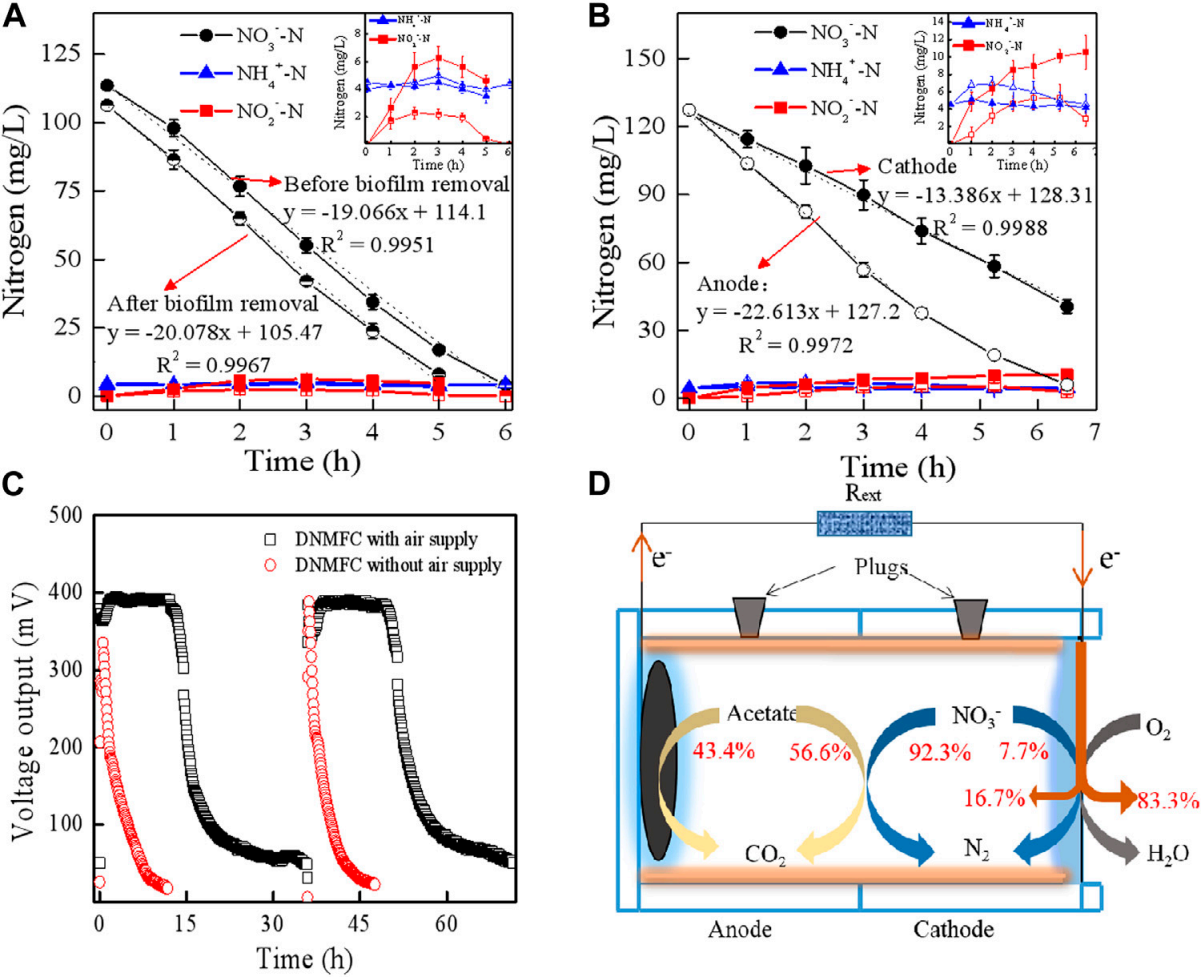
Contributions of nitrate reduction in electron consumption and acceptance in DNMFCs at the COD/NO_3_^−^-N ratio of 7. **(A)** Solid are the nitrogen concentrations in the DNMFC before biofilm removal, and semi-solid are those after biofilm removal. **(B)** Solid are the nitrogen concentrations in the cathode chamber of the DC-DNMFC, and hollow are those in the anode chamber of the DC-DNMFC. **(C)** Voltage output in the DNMFC with or without air. **(D)** Distributions of acetate, nitrate, and electron in the single-chambered DNMFC.

Considering the voltage waves, electrode potentials, DO, and COD, DNMFCs with a single chamber and two chambers exhibited similar behaviors under the same operational conditions, which could be due to the minimal effects exerted by protons and oxygen on the electricity performance (Supplementary Figure S2). Furthermore, the biofilm growing on the wall also slightly affected the denitrification because there was no significant difference in the nitrate removal rate after removing the biofilms from the DNMFCs (Figure 3A). Additionally, a high concentration of NO_2_^−^-N was observed before the biofilm removal, implying that the intermediate accumulation was mainly attributed to the process of traditional biological denitrification (Sun et al., 2010). As shown in Figure 3B, different trends of nitrogen removal rate were observed in the anode and cathode chambers of DC-DNMFCs. Considering NO_3_^−^-N removal rates, the mean value in the anode chamber (22.6 mg/L/h) was considerably higher than that in the cathode chamber (13.4 mg/L/h), demonstrating that the high abundance of electroactive biofilm promotes anode heterotrophic denitrification by extracellular electron transfer, as previously described (Tong and He, 2013; Sotres et al., 2016). NO_2_^−^-N accumulation in the cathode chamber was notably greater than that in the anode chamber, verifying that DNB-mediated biological denitrification produced more intermediates than that produced by EDNB (Tong and He, 2013). Nitrous oxide (N_2_O) as an intermediate product of biological denitrification is significant for its strong greenhouse gas effect and has been detected in our previous studies (Jin et al., 2022). However, the percentage of residual gaseous N_2_O was less than 0.0001%, which can be ignored during the mass conversion of nitrate reduction in DNMFCs by heterotrophic denitrification.

The distribution of acetate, nitrate, and electrons in DNMFCs changed with the COD/NO_3_^−^-N ratios. In this system, we designed a series of experiments for calculating the proportion of each reaction at a COD/NO_3_^−^-N ratio of 7. For example, the hollow slab at the air side of the cathode was replaced with a solid plate to block the air, which can calculate the generated electrons accepted by nitrate. The results showed that the voltages of DNMFCs without air supply sharply decreased, and the time for a complete cycle lasted less than 10 h (Figure 3C). As shown in Supplementary Table S1, NO_3_^−^-N removal remained unaffected in both conditions of air supply and no air supply. However, COD removal sharply declined, and only approximately 7.8 μmol/e of electrons were recovered by DNMFCs without air supply. The value of E_N_ should be 7.8 μmol/e; therefore, the concentration of NO_3_^−^-N reduced by electrochemical denitrification was 7.5 mg/L using Eq. 2, which accounted for 7.7% of the original concentration. The residual nitrate level of 92.3% was reduced by heterotrophic denitrification. Conversely, the voltage of DNMFCs with air supply maintained a stable output of 400 mV in 15 h, and the entire cycle lasted for more than 35 h, implying that ORR was still dominant in the air-cathode of DNMFCs. Approximately 46.7 μmol/e of electrons were recovered in DNMFCs with air supply. Therefore, in DNMFCs, nitrate as an electron acceptor only accounted for 16.7% of all collected electrons, and the other electrons were accepted by oxygen. Similarly, the amount of COD from the number of collected electrons could be calculated as 107.2 mg/L using Eq. 3. The theoretical consumed COD for electricity generation can be calculated, assuming that a CE of 38.0% is constant in DNMFCs. The results showed that 43.4% of COD was used for power recovery, and the rest was used for heterotrophic denitrification. Above all, the distributions of acetate, nitrate, and electron in DNMFCs at the COD/NO_3_^−^-N ratio of 7 are shown in Figure 3D.

### 3.3 Microbial community analysis of biofilms

Nitrate stimulates the growth of nitrogen-metabolizing bacteria, resulting in DNB enrichment, with a remarkable ecological impact on DNMFCs (Huang et al., 2018). *Azonexus* and *Pseudomonas*, which are known as DNB and EDNB, prefer to grow in nitrogen-containing solutions, becoming the dominant species in DNMFCs (Jangir et al., 2016; Yun et al., 2018; Yang et al., 2019). DC-DNMFCs can be considered the transition state of the dynamic development in DNMFCs. The biomass density of electrode biofilms of DNMFCs considerably differed when viewed using an SEM and a CLSM (Supplementary Figure S3). During long-term operations, copious amounts of bacteria in the inner layer of the electrode biofilms died, demonstrating that the inner layer of the thick biofilm cannot satisfy the nutrient demand in the oligotrophic micro-environment (Yuan et al., 2021).

**FIGURE 4:**
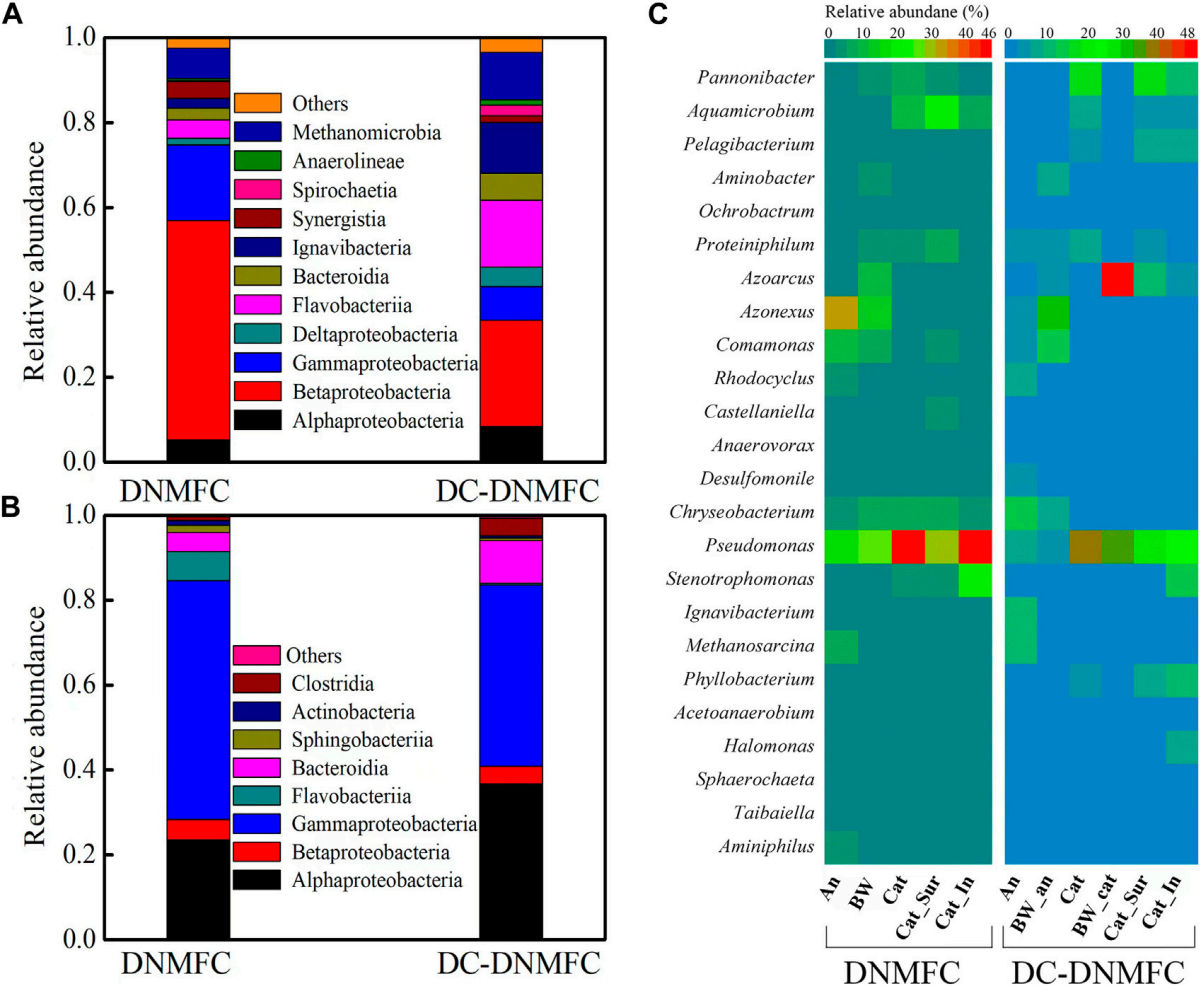
Microbial community analysis of the anodes **(A)** and cathodes **(B)** in DNMFCs at the class level and the genus level heatmap **(C)** in different positions (An and BW are the biomass from the anode and the biofilm on the wall, while Cat, Cat_Sur, and Cat_In are the biomass from the total cathode, the surface biofilm of the cathode, and the inner biofilm of the cathode, respectively). The heatmap contains the relative abundance of the species, which is more than 1% in all samples.

The microbial community structure at the class level (abundance >1%) and the heatmap of their bacterial distribution at the genus level (abundance >1%) between DNMFCs and DC-DNMFCs were analyzed, as shown in Figures 4A–C. At the class level, Alphaproteobacteria and Gammaproteobacteria accounted for 51.6% and 17.8%, respectively, in the anode biofilm of DNMFCs, which decreased to 25.0% and 7.9% in the DC-DNMFCs. In contrast, Flavobacteriia and Ignavibacteria remarkably increased in the anode of DC-DNMFCs. Compared to the anode, the gap between microbial communities was closer in the cathode between DNMFCs and DC-DNMFCs, and the relative abundances of Proteobacteria (including Alphaproteobacteria, Betaproteobacteria, and Gammaproteobacteria) reached 84.5% and 83.5%, respectively. Moreover, Flavobacteriia dominated the cathode of DNMFCs, while Bacteroidia dominated the DC-DNMFCs. At the genus level, the dominant genera of anode biofilm in DNMFCs were in the order of *Azonexus*, *Pseudomonas*, *Comamonas*, *Chryseobacterium*, and *Aminiphilus*, which are considered DNB and EAB (Figure 4C). However, biofilms attached to the anode of DC-DNMFCs noticeably enriched the sequences related to *Chryseobacterium*, *Ignavibacterium*, *Rhodocyclus*, and *Pseudomonas*. *Pseudomonas* accounted for 49.6% of the DNMFCs and 40.4% of the DC-DNMFCs in cathode biofilms, respectively. The high abundance absolutely dominated the cathode microbial community, indicating that this special genus remarkably reduced nitrate by heterotrophic/electrochemical denitrification (Li et al., 2019). The genus *Aquamicrobium* exhibited a remarkable similarity between DNMFCs and DC-DNMFCs, which might relate to the original inoculum. Moreover, the genus *Pannonibacter* (Sun et al., 2016), a new type of denitrifier, was also enriched in DNMFCs, which dramatically increased in DC-DNMFCs.

Additionally, the microbial community compositions of biofilms growing on the walls of the entire DCMFC, the DC-DNMFC anode chamber, and the DC-DNMFC cathode chamber were also investigated to understand the dynamic rule of bacterial change. Meanwhile, the cathode biofilms, divided into the surface and inner layers, were also analyzed at the genus level. The former can be easily washed away by running water, and the latter tightly adheres to the cathode. Furthermore, the proportions of special genera significantly differed on the anode, wall, cathode, cathode surface layer, and cathode inner layer. For example, the proportions of the dominant genus *Pseudomonas* distributed in the DNMFCs were 17.6% (anode), 27.8% (wall), 49.6% (cathode), 30.6% (surface layer), and 49.8% (inner layer). This genus grew majorly on both the electrodes and wall, which were maintained at high levels in DNMFCs. In the DC-DNMFCs, the percentages of *Pseudomonas* changed to 7.3% (anode), 6.5% (anode wall), 40.4% (cathode), 35.9% (cathode wall), 27.6% (surface layer), and 25.3% (inner layer). These results indicate that *Pseudomonas* prefers a micro-aerobic environment of cathode for growth, although it presents high activity for nitrate reduction under a strictly anaerobic environment of anode. Few aerobic bacteria (including *Aquamicrobium* and *Azoarcus*) exhibited remarkably high relative abundance in the cathode chamber, consuming O_2_ permeated from the air-cathode, thereby making the cathode a hypoxic environment suitable for the growth of denitrifiers. In contrast, the genus *Azonexus* dominated the anode of DNMFCs with a high relative abundance of 33.1%, which practically disappeared in the cathode biofilm, implying that *Azonexus* as an alternative DNB needs anaerobic conditions for growth. The proportions of *Azonexus* in DC-DNMFCs at the anode and the wall of the anode chamber accounted for 5.8% and 32.8%, respectively, which is a critical proof to verify the above speculations. Furthermore, functional genera consisted of *Proteiniphilum*, *Comamonas*, *Rhodocyclus*, *Chryseobacterium*, and *Aminobacter*. Moreover, other genera with high percentages in the anode biofilm and low proportions in the wall could be considered EAB, which included *Rhodocyclus*, *Desulfomonile*, and *Ignavibacterium*. Additionally, *Methanosarcina*, methane-producing bacteria, also increased to 11.1% at the anode of DC-DNMFCs, compared to 7.2% at the anode of DNMFCs. These results were consistent with the fact that DNB and EAB could be dominant in the MFC-based wastewater treatment process (Yang et al., 2020; Yang et al., 2021).

### 3.4 Ecological contribution of DNMFCs

In this study, functional bacteria can be grouped into three categories: DNB, EAB, and EDNB. First, DNB were rapidly accumulated in the solution and then adhered to the reactor walls or adsorbed onto the electrodes (anaerobes on the anode and facultative anaerobes on the cathode). DNB with low electron transfer capacity, such as *Azonexus*, *Azoarcus*, and *Comamonas*, possessed low relative abundances in the electrode biofilm but high abundances in the wall biofilm. Second, EAB that could adapt to the nitrate-containing aqueous environment were either retained or gradually eliminated during the system operation. The relative abundances of EAB, such as *Ignavibacterium*, *Rhodocyclus*, and *Desulfomonile*, except for *Geobacter*, notably increased in DC-DNMFCs, indicating that these genera were likely to have a positive response to nitrate exposure (Yang et al., 2021). Finally, the relative abundance of *Pseudomonas* increased considerably at both the cathode and anode biofilms, which identified its capacity to metabolize nitrogen and transfer electrons. Previous studies have confirmed that *Pseudomonas* is widespread in the electrode, which contains nitrate-reducing species (Ilamathi et al., 2019). In this system, *Pseudomonas* may be related to simultaneous nitrate reduction and electron transfer, which was consistent with the previous study (Jin et al., 2022).

**FIGURE 5:**
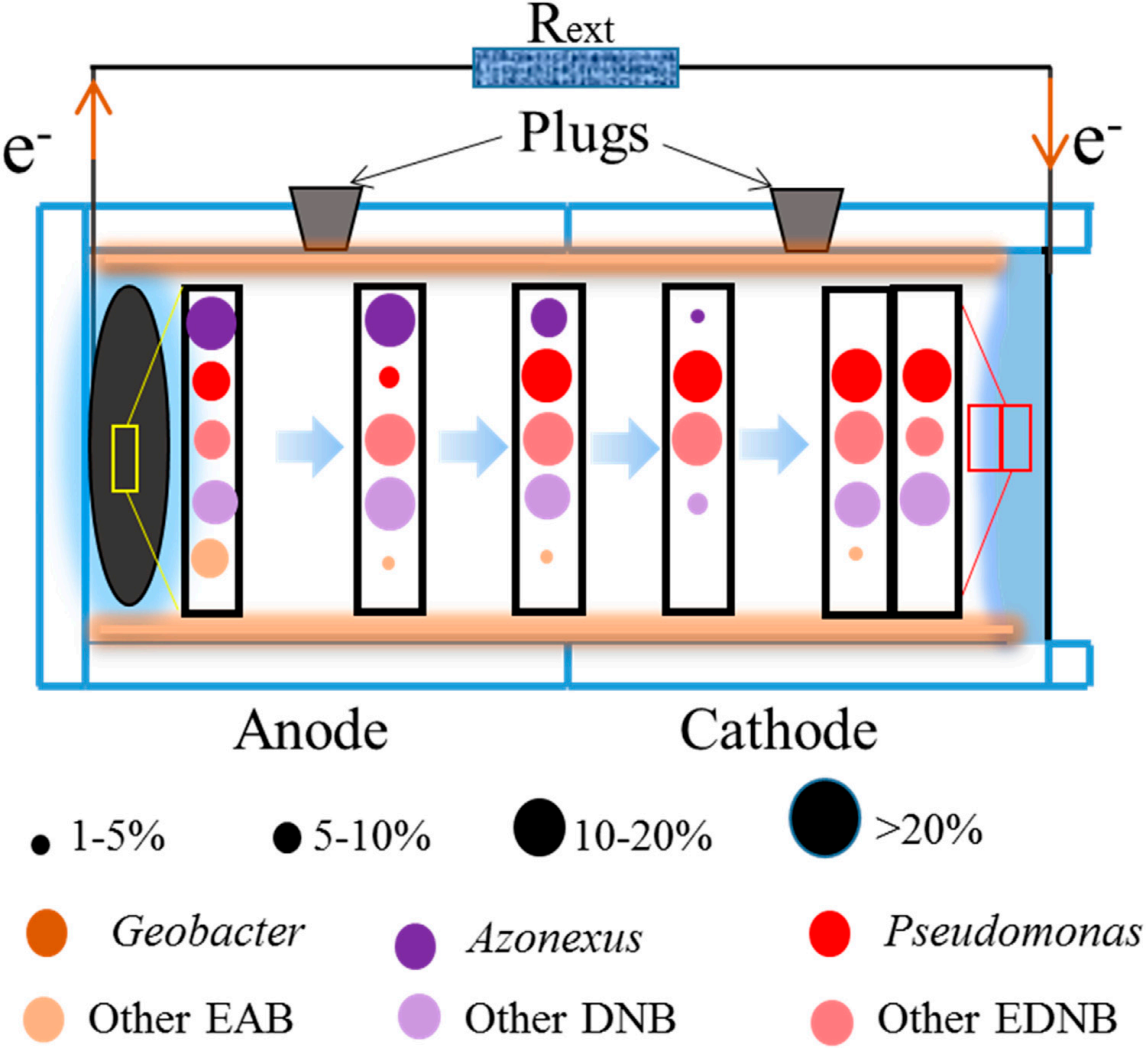
Change trends in the functional bacteria in DNMFCs, especially the distribution of the dominant genera.

According to the taxonomy information, the evolution process of the dominant functional bacteria from anode to cathode, especially the distribution of the dominant genera in DNMFCs, is illustrated in Figure 5. *Azonexus* and EAB were noticeably enriched in the anode chamber, which gradually weakened until they completely disappeared at the cathode, indicating that the bacteria are strictly anaerobic. However, other DNB, mostly known as facultative anaerobes, grew throughout the reactor with increased relative abundances. Furthermore, DNMFC systems are believed to favor the growth of EAB with nitrate-reducing ability, such as *Pseudomonas*, which was considered the most remarkable contributor, absolutely dominating nitrate-containing wastewater treatment (Sun et al., 2016; Yang et al., 2020). Therefore, nitrate exposure not only influences the microbial community structure but also enriches the relative functional bacteria, resulting in the increase in an cathode biofilm (Li et al., 2019; Guo and Liu, 2020).

## 4 Conclusion

This study comprehensively compared the performance of DNMFCs with NH_4_^+^- and NO_3_^−^-containing wastewater treatment and revealed the distribution of nutrients and functional bacteria. DNMFCs without nitrifying processes exhibited stable electrochemical activity and high nitrate removal efficiency. In DNMFCs with C/N = 7, 56.6%, acetate was used for heterotrophic denitrification, and the residual was used to generate electricity; meanwhile, 92.3% of nitrate was removed by heterotrophic denitrification, and the rest was reduced via electrochemical denitrification. The distribution rule of the dominant functional bacteria in DNMFCs was EAB in the anode, anaerobic DNB in the solution, facultative anaerobic DNB in the solution and cathode, and EDNB in the entire reactor. In a word, this study provides theoretical guidance for the practical application of DNMFCs in nitrogen-containing wastewater treatment and online water quality monitoring for organic or nitrogen cycling in the environment.

## Data Availability

The original contributions presented in the study are included in the article/Supplementary Material; further inquiries can be directed to the corresponding author.

## References

[B1] American Public Health Association (APHA) (2001). Standard methods for examination of water and wastewater. 21. Washington DC, USA: Water Environment Federation.

[B2] Gao Y. Y. Wang S. Yin F. J. Hu P. Wang X. Z. Liu Y. (2021). Enhancing sensitivity of microbial fuel cell sensors for low concentration biodegradable organic matter detection: regulation of substrate concentration, anode area and external resistance. J. Environ. Sci. 101, 227–235. 10.1016/j.jes.2020.08.020 33334518

[B3] Guo F. Liu H. (2020). Impact of heterotrophic denitrification on BOD detection of the nitrate–containing wastewater using microbial fuel cell -based biosensors. Chem. Eng. J. 394, 125042. 10.1016/j.cej.2020.125042

[B4] Gurung A. Thapa B. S. Ko S.-Y. Ashun E. Toor U. A. Oh S.-E. (2023). Denitrification in microbial fuel cells using granular activated carbon as an effective biocathode. Energies 16 (2), 709. 10.3390/en16020709 38307327

[B5] Huang H. B. Cheng S. A. Yang J. W. Li C. C. Sun Y. Cen K. F. (2018). Effect of nitrate on electricity generation in single-chamber air cathode microbial fuel cells. Chem. Eng. J. 337, 661–670. 10.1016/j.cej.2017.12.150

[B6] Ilamathi R. Sheela A. M. Gandhi N. N. (2019). Comparative evaluation of *Pseudomonas* species in single chamber microbial fuel cell with manganese coated cathode for reactive azo dye removal. Int. Biodeterior. Biodegr. 144, 104744. 10.1016/j.ibiod.2019.104744

[B7] Jangir Y. French S. Momper L. M. Moser D. P. Amend J. P. El-Naggar M. Y. (2016). Isolation and characterization of electrochemically active subsurface delftia and *Azonexus* species. Front. Microbiol. 7, 756. 10.3389/fmicb.2016.00756 27242768 PMC4876122

[B8] Jin X. J. Yang N. Liu H. Wang S. (2022). Membrane penetration of nitrogen and its effects on nitrogen removal in dual-chambered microbial fuel cells. Chemosphere 297, 134038. 10.1016/j.chemosphere.2022.134038 35183587

[B9] Knowles R. (1982). Denitrification. Rev. 46 (1), 43–70. 10.1128/mr.46.1.43-70.1982 PMC373209 7045624

[B10] Li Z. H. Zhang Q. H. Jiang Q. R. Zhan G. Q. Li D. P. (2019). The enhancement of iron fuel cell on bio-cathode denitrification and its mechanism as well as the microbial community analysis of bio-cathode. Bioresour. Technol. 274, 1–8. 10.1016/j.biortech.2018.11.070 30496969

[B11] Qiao S. Yin X. Zhou J. T. Wei L. E. Zhong J. Y. (2018). Integrating anammox with the autotrophic denitrification process via electrochemistry technology. Chemosphere 195, 817–824. 10.1016/j.chemosphere.2017.12.058 29289909

[B12] Sotres A. Cerrillo M. Vinas M. Bonmati A. (2016). Nitrogen removal in a two-chambered microbial fuel cell: establishment of a nitrifying-denitrifying microbial community on an intermittent aerated cathode. Chem. Eng. J. 284, 905–916. 10.1016/j.cej.2015.08.100

[B13] Sun Q. Li Z. L. Wang Y. Z. Yang C. X. Chung J. S. Wang A. J. (2016). Cathodic bacterial community structure applying the different co-substrates for reductive decolorization of Alizarin Yellow R. Bioresour. Technol. 208, 64–72. 10.1016/j.biortech.2016.02.003 26922314

[B14] Sun S.-P. Nàcher C. P. i. Merkey B. Zhou Q. Xia S.-Q. Yang D.-H. (2010). Effective biological nitrogen removal treatment processes for domestic wastewaters with low C/N ratios: a review. Environ. Eng. Sci. 27 (2), 111–126. 10.1089/ees.2009.0100

[B15] Tong Y. R. He Z. (2013). Nitrate removal from groundwater driven by electricity generation and heterotrophic denitrification in a bioelectrochemical system. J. Hazard. Mater. 262, 614–619. 10.1016/j.jhazmat.2013.09.008 24096001

[B16] Yan H. J. Saito T. Regan J. M. (2012). Nitrogen removal in a single-chamber microbial fuel cell with nitrifying biofilm enriched at the air cathode. Water Res. 46 (7), 2215–2224. 10.1016/j.watres.2012.01.050 22386083

[B17] Yang J. W. Cheng S. A. (2019). Effects of using anode biofilm and cathode biofilm bacteria as inoculum on the start-up, electricity generation, and microbial community of air-cathode single-chamber microbial fuel cells. Pol. J. Environ. Stud. 28 (2), 693–700. 10.15244/pjoes/81700

[B18] Yang N. Liu H. Zhan G. Q. Li D. P. (2020). Sustainable ammonia-contaminated wastewater treatment in heterotrophic nitrifying/denitrifying microbial fuel cell. J. Clean. Prod. 245, 118923. 10.1016/j.jclepro.2019.118923

[B19] Yang N. Zhan G. Q. Li D. P. Wang X. He X. H. Liu H. (2019). Complete nitrogen removal and electricity production in Thauera-dominated air-cathode single chambered microbial fuel cell. Chem. Eng. J. 356, 506–515. 10.1016/j.cej.2018.08.161

[B20] Yang N. Zhou Q. M. Zhan G. Q. Liu Y. L. Luo H. Q. Li D. P. (2021). Comparative evaluation of simultaneous nitritation/denitritation and energy recovery in air-cathode microbial fuel cells (ACMFCs) treating low C/N ratio wastewater. Sci. Total Environ. 788, 147652. 10.1016/j.scitotenv.2021.147652 34023598

[B21] Yuan J. Q. Yuan H. G. Huang S. B. Liu L. J. Fu F. C. Zhang Y. Q. (2021). Comprehensive performance, bacterial community structure of single-chamber microbial fuel cell affected by COD/N ratio and physiological stratifications in cathode biofilm. Bioresour. Technol. 320, 124416. 10.1016/j.biortech.2020.124416 33220541

[B22] Yun H. Liang B. Kong D. Y. Wang A. J. (2018). Improving biocathode community multifunctionality by polarity inversion for simultaneous bioelectroreduction processes in domestic wastewater. Chemosphere 194, 553–561. 10.1016/j.chemosphere.2017.12.030 29241129

[B23] Zhang F. He Z. (2012). Integrated organic and nitrogen removal with electricity generation in a tubular dual-cathode microbial fuel cell. Process Biochem. 47 (12), 2146–2151. 10.1016/j.procbio.2012.08.002

[B24] Zhang F. Saito T. Cheng S. A. Hickner M. A. Logan B. E. (2010). Microbial fuel cell cathodes with poly(dimethylsiloxane) diffusion layers constructed around stainless steel mesh current collectors. Environ. Sci. Technol. 44 (4), 1490–1495. 10.1021/es903009d 20099808

[B25] Zhang R. Wu Y. Wang L. T. Wu Q. Zhang H. W. (2020). Cathode denitrification of microbial fuel cells. Prog. Chem. 32 (12), 2013–2021. 10.7536/PC200332

