# Supplementary material for: Insight into a single-chamber air-cathode microbial fuel cell for nitrate removal and ecological roles
Source: Front Bioeng Biotechnol. 2024 Jul 8;12:1397294. doi: 10.3389/fbioe.2024.1397294 (PMC11260741; doi:10.3389/fbioe.2024.1397294)
Supplement: Supplementary file 1 [file DataSheet1.PDF]

Supplementary Information

Insight into a single chamber denitrifying microbial fuel cell for nitrate removal and ecological roles

Xiaojun Jin [1, 2], Nuan Yang [3], Dake Xu [1], Zhuo Yan[4], Cheng Song [2], Hong Liu [2*]

[1] Shenyang National Laboratory for Materials Science, Northeastern University, Shenyang 110819, China

[2] CAS Key Laboratory of Reservoir Aquatic Environment, Chongqing Institute of Green and Intelligent Technology, Chinese Academy of Sciences, Chongqing 400714, China

[3] Key Laboratory of Development and Application of Rural Renewable Energy, Ministry of Agriculture and Rural Affairs, Biogas Institute of Ministry of Agriculture and Rural Affairs (BIOMA), Chengdu 610041, China

[4] Department of Artificial Intelligence, Shenyang Aerospace University, Shenyang 110136, China

* Correspondence:

Hong Liu

liuhong@cigit.ac.cn

The authors declare no competing financial interest.

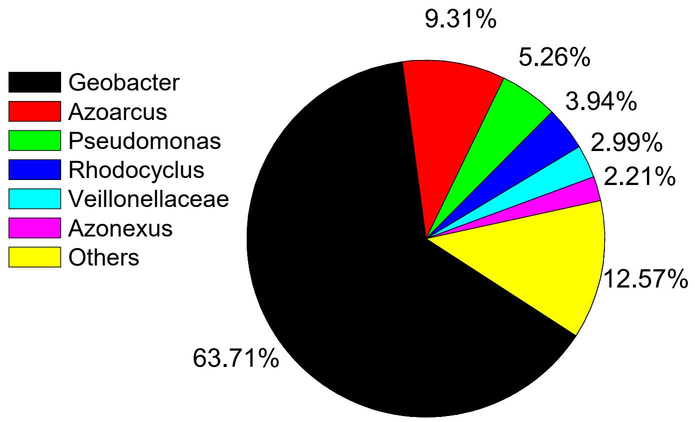

Figure S1. Microbial community composition of the anodic biofilm in the large-scale MFC at the genus level

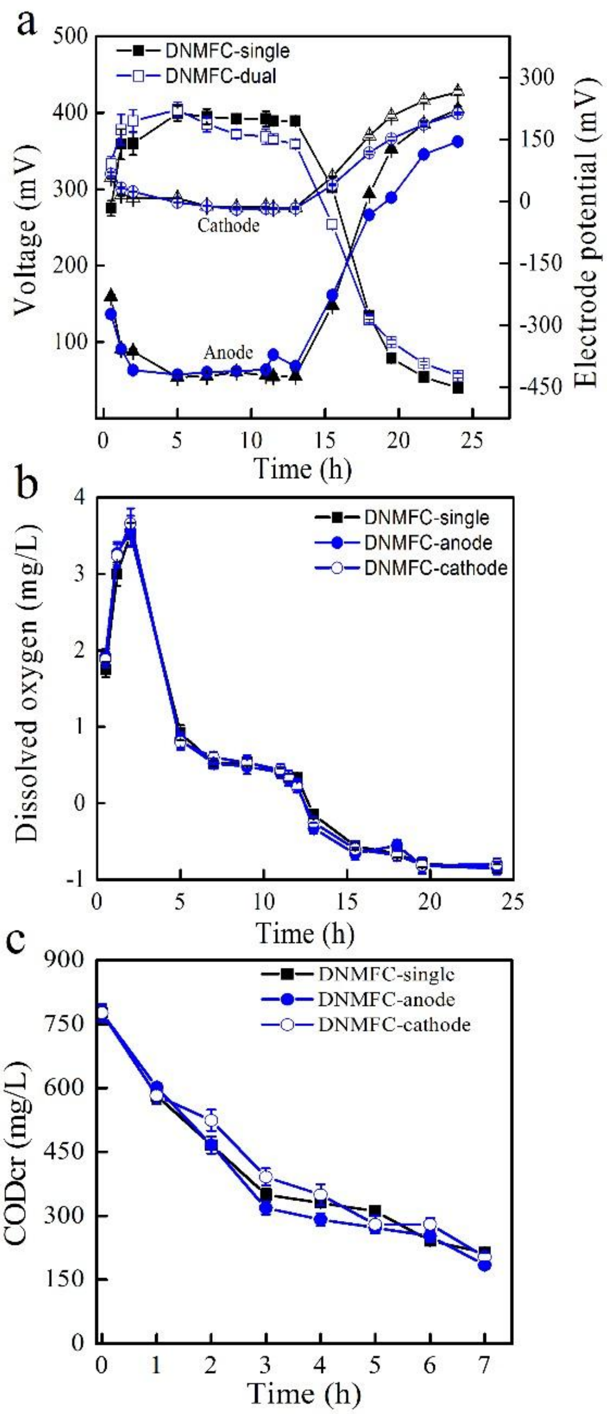

Figure S2. Comparison of the potentials, DO and COD between DNMFC with single chamber and dual chambers. Solid are the single-chamber DNMFC, hollow are the dual-chamber DNMFC.

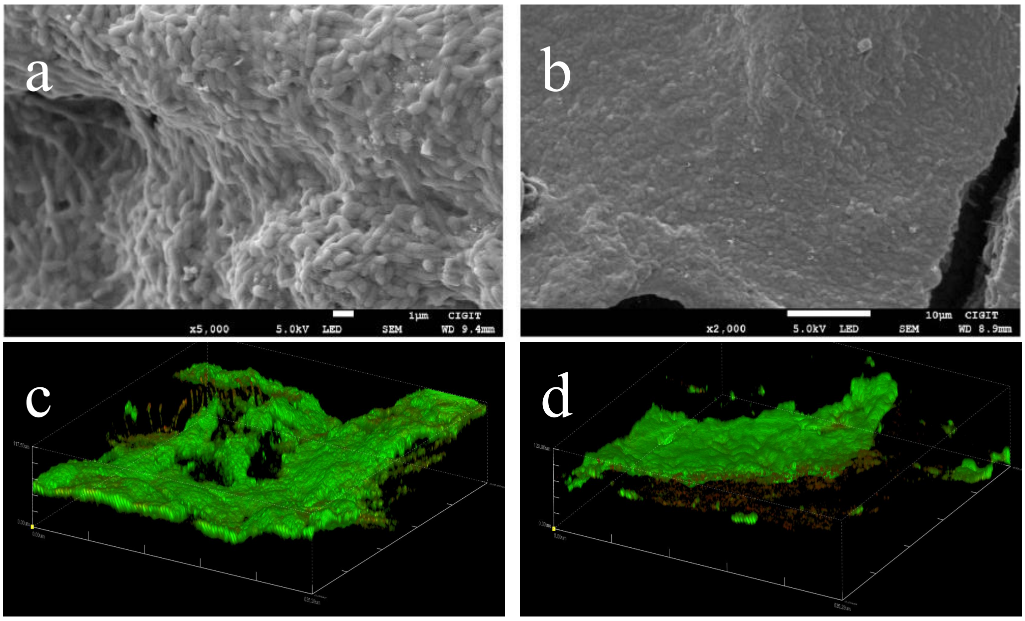

Figure S3. SEM and CLSM of electrodes in the DNMFCs after 50 days operation. (a) and (c) are the anodes, (b) and (d) are the cathodes.

Table S1 Comparisons of energy recovery, COD removal and nitrogen removal in the single-chamber DNMFC and dual-chamber DNMFC with/without air supply.

	DNMFC		DC-DNMFC			
	Air-cat	No-air	An_1	Air-cat	An_2	No-air
Electron recovery (NO_3^-)	46.7±0.2	7.8±0.1	56.4±0.2		7.2±0.1	
COD removal (%)	95.4±0.2	57.9±0.1	93.6±0.3	87.6±1.0	64.1±1.4	54.9±1.8
NO_3^--N removal (%)	99.2±1.0	99.1±0.1	99.2±0.6	98.8±0.5	98.8±0.2	98.9±0.2
NO_2^--N generation (%)	0.03±0.01	0.04±0.01	0.04±0.01	0.03±0.01	0.03±0.01	0.05±0.01
NH_4^+-N generation (%)	1.1±0.2	4.6±0.1	4.4±0.1	3.7±0.1	5.5±0.1	4.3±0.1
TN removal (%)	97.1±0.3	94.5±0.2	94.7±0.2	95.1±0.1	93.2±0.2	94.5±0.1
